# Laccase Catalyzed Synthesis of Iodinated Phenolic Compounds with Antifungal Activity

**DOI:** 10.1371/journal.pone.0089924

**Published:** 2014-03-03

**Authors:** Julian Ihssen, Mark Schubert, Linda Thöny-Meyer, Michael Richter

**Affiliations:** 1 Laboratory for Biomaterials, Empa, Swiss Federal Laboratories for Materials Science and Technology, St. Gallen, Switzerland; 2 Laboratory for Applied Wood Materials, Empa, Swiss Federal Laboratories for Materials Science and Technology, St. Gallen, Switzerland; University of Wisconsin - Madison, United States of America

## Abstract

Iodine is a well known antimicrobial compound. Laccase, an oxidoreductase which couples the one electron oxidation of diverse phenolic and non-phenolic substrates to the reduction of oxygen to water, is capable of oxidizing unreactive iodide to reactive iodine. We have shown previously that laccase-iodide treatment of spruce wood results in a wash-out resistant antimicrobial surface. In this study, we investigated whether phenolic compounds such as vanillin, which resembles sub-structures of softwood lignin, can be directly iodinated by reacting with laccase and iodide, resulting in compounds with antifungal activity. HPLC-MS analysis showed that vanillin was converted to iodovanillin by laccase catalysis at an excess of potassium iodide. No conversion of vanillin occurred in the absence of enzyme. The addition of redox mediators in catalytic concentrations increased the rate of iodide oxidation ten-fold and the yield of iodovanillin by 50%. Iodinated phenolic products were also detected when *o*-vanillin, ethyl vanillin, acetovanillone and methyl vanillate were incubated with laccase and iodide. At an increased educt concentration of 0.1 M an almost one to one molar ratio of iodide to vanillin could be used without compromising conversion rate, and the insoluble iodovanillin product could be recovered by simple centrifugation. The novel enzymatic synthesis procedure fulfills key criteria of green chemistry. Biocatalytically produced iodovanillin and iodo-ethyl vanillin had significant growth inhibitory effects on several wood degrading fungal species. For *Trametes versicolor*, a species causing white rot of wood, almost complete growth inhibition and a partial biocidal effect was observed on agar plates. Enzymatic tests indicated that the iodinated compounds acted as enzyme responsive, antimicrobial materials.

## Introduction

There is a continuing need for effective antifungal compounds, preferentially derived from renewable resources, which at the same time pose low risks to human health and the environment. Elemental iodine (I_2_) is a well-known antiseptic and disinfectant that acts against bacteria, fungi and viruses at millimolar concentrations [1]. The biocidal mechanism is thought to involve the modification of nucleotides, fatty acids, as wells as cysteine and methionine groups of proteins [1]. Molecular iodine (I_2_) can only be dissolved in water in the presence of a two-fold molar excess of iodide ions (I^-^) which facilitate the formation of water-soluble triiodide (I_3_^−^, [I-I-I]^−^). This type of formulation is also called Lugol's solution. In fact, in aqueous iodine solutions at least seven iodine species co-exist in a complex equilibrium, making such systems inherently unstable [1]. The drawbacks of aqueous iodine solutions such as instability, skin staining and irritation can be overcome by the use of iodine-binding and releasing polymers (iodophores) such as polyvinylpyrrolidone [1]. Certain organo-iodine compounds, e.g. iodopropynyl butylcarbamate [2] are effective, widely used antifungal agents.

Interestingly, the enzyme laccase is capable of directly oxidizing iodide to iodine (I_2_) in aqueous solution, which in turn is in equilibrium with triiodide [3]. Laccases are type III multicopper oxidases which oxidize numerous aromatic and non-aromatic compounds [4]. The electrons generated by substrate oxidation are transferred to molecular oxygen, which is reduced to H_2_O without any further byproducts [5]. In contrast to peroxidases, laccases do not require hydrogen peroxide as co-substrate. Due to their high specific activity, stability and broad substrate range, laccases are increasingly applied in organic synthesis [4]. Enzymatic iodide oxidation was demonstrated for several fungal laccases and recently also for a bacterial multicopper oxidase [3], [6], [7]. The use of redox mediators such as 2,2′-azino-bis(3-ethylbenzthiazoline-6-sulphonic acid) (ABTS) or methyl syringate was shown to increase iodide oxidation rates considerably [3], [7]. The iodide-oxidizing activity of laccase was used for creating paints containing immobilized enzyme, which render surfaces bactericidal and sporicidal upon application of iodide solution [8]. In a previous study we demonstrated that the treatment of conifer wood with laccase and iodide, either with or without redox mediators, generated a wash-out resistant, antibacterial and antifungal surface [9]. In contrast to iodide-only treatment, laccase-iodide functionalized, leached wood prevented growth of basidiomycetes, causing white and brown rot, and of ascomycetes, causing blue stain discoloration of wood. FTIR analysis indicated that the chemical structure of the phenolic (lignin) part of the wood structure was modified in a specific way by the reaction with laccase and iodide.

Due to the observed antifungal effect obtained by laccase-iodide treatment of wood we hypothesized that wood-related aromatic molecules can be directly iodinated in the presence of unreactive iodide by laccase catalysis, yielding compounds with potential antifungal activity. Such biotransformations, which have not been investigated previously, might also be generally useful for the eco-friendly synthesis of iodinated compounds. A recent study suggested that laccase-catalyzed oxidation of iodide and subsequent formation of organically bound iodine can occur in natural environments [10]. Electrophilic iodine species such as I_2_ and hypoiodous acid (HIO) were proposed to react with aromatic rings of soil organic matter [11], indicating that a similar reaction of laccase-generated reactive iodine species with lignin-derived phenolic compounds is possible.

## Materials and Methods

### Chemicals and enzymes

Potassium iodide (KI), 2,2′-azino-bis(3-ethylbenzthiazoline-6-sulphonic acid) (ABTS), 3′,5-dimethoxy-4′-hydroxyacetophenone (acetosyringone, ACS), 4-hydroxy-3-methoxybenzaldehyde (vanillin), 5-iodovanillin, 2-hydroxy-3-methoxybenzaldehyde (*o*-vanillin), 4-hydroxy-3-ethoxybenzaldehyde (ethyl vanillin), 4′-hydroxy-3′-methoxyacetophenone (acetovanillone), methyl 4-hydroxy-3-methoxybenzoate (methyl vanillate), 4-hydroxy-3-methoxybenzoic acid (vanillic acid), 4-hydroxy-3-methoxybenzyl alcohol (vanillyl alcohol), α-naphthol, pyrogallol, malt extract agar, buffer components and solvents were purchased from Sigma-Aldrich (Buchs, Switzerland) in standard reagent grade. Freeze-dried preparations of *Trametes versicolor* laccase were purchased from Sigma-Aldrich and stored at −20°C before use. Activity of the commercial laccase preparations was verified with the substrate ABTS at pH 4 and room temperature and always ranged from 20 to 30 U mg^−1^.

### Biochemical assays

Laccase catalyzed iodide oxidation was monitored by measuring the formation of brownish colored triiodide species at 353 nm in 0.2 M citrate-phosphate buffer (McIlvain) at pH 5.0 and room temperature. An extinction coefficient for I_3_^−^ of 4.65×10^3^ M^−1^ cm^−1^ was determined by titrating iodine to 50 mM KI in the same buffer. This extinction coefficient is close to the value of 4.53×10^3^ M^−1^ cm^−1^ reported by Kulys *et al.* for pH 5.5 [7]. One unit was defined as the amount of enzyme that lead to the formation of 1 µmol triiodide per minute. The pH optimum for iodide oxidation with and without mediators (10 µM) was determined by measuring relative rates of triiodide formation in a McIlvain buffer series (0.1–0.2 M citric acid-potassium phosphate, pH 2.2 to 8.0 in 0.2 steps) at room temperature with a laccase concentration of 0.25 mg mL^−1^ and an iodide concentration of 50 mM. The kinetics of iodide oxidation were determined within a concentration range of 0–200 mM KI with and without mediators (ABTS and ACS, 10 µM) or second substrates (vanillin 100 µM) in the presence of either 0.01 (mediators ABTS, ACS) or 0.1 (vanillin, no mediator) mg mL^−1^ *T. versicolor* laccase in McIlvain buffer pH 5. Apparent kinetic parameters (*V*_max_, *K*_M_ and *K*_i_) for iodide oxidation were calculated from initial rates (4 min) by non-linear regression algorithms according to the Michaelis-Menten equation with substrate inhibition using SIGMA-PLOT Enzyme Kinetics software. Laccase activity at the begin and end of the biotransformations was determined with ABTS (5 mM) as substrate in tartaric acid buffer (50 mM) at pH 4.0 and room temperature (extinction coefficient for oxidized ABTS at 420 nm: 36×10^3^ M^−1^ cm^−1^, [12]).

### Biotransformations

Small scale biotransformations and control reactions were carried out in 15 mL closed plastic tubes with 1 or 2 mL liquid volume which were incubated horizontally and light-protected on a shaker with an agitation of 150 rpm for 20 h at room temperature. Reactions were carried out in 0.2 M citrate-phosphate buffer (McIlvain) at pH 5.0 and *T. versicolor* laccase was added to a final concentration of 0.25 mg mL^−1^ (4 U mL^−1^, ABTS, pH 4). Phenolic compounds were added to a final concentration of 4 mM as 20-fold concentrated stock solutions (solvent: ethanol, final ethanol concentration 5% v/v) and KI was added in 12.5-fold molar excess (50 mM). In a subset of experiments ABTS or ACS were added in catalytic concentrations (100 µM) as redox mediators. Laccase-free control reactions were always performed in parallel with biotransformations. Reaction products were extracted by addition of 1 to 2 mL ethyl acetate. The time course of laccase-catalyzed iodination was recorded for similar reactions carried out in open 500 mL Erlenmeyer flasks (reaction batch volume 50 mL). Samples for HPLC-MS were taken in regular intervals and extracted with ethyl acetate. The triiodide concentration was measured in the ethyl acetate phase at 353 nm because brownish colored compounds could be completely extracted from the aqueous phase with this solvent. Prior to sample storage and HPLC-MS measurements, triiodide was removed from ethyl acetate extracts by mixing/washing with 50% w/v potato starch solution in water until the color had been reduced to light brown.

For preparative biotransformations, magnetically stirred Erlenmeyer flasks with a total volume of 2 L and a liquid volume of 1 L were used. Flasks were aerated with pressurized air through a frit. Reaction mixtures contained 4 mM of either vanillin or ethyl vanillin, 0.25 mg mL^−1^ *T. versicolor* laccase, 100 µM ABTS and 50 mM KI in McIlvain buffer pH 5 with 5% v/v ethanol. Reaction products were extracted after 20 h of incubation at room temperature by addition of 250 mL ethyl acetate, thorough mixing and subsequent separation of the organic phase. The solvent was removed in a rotary evaporator at 50°C under vacuum until solid products were obtained. For determination of purity, reaction products were re-dissolved in ethyl acetate to a concentration of 2 mg mL^−1^ and analyzed by HPLC-MS.

Vanillin biotransformations at high substrate concentrations were performed in 100 mL Erlenmeyer flasks in a total liquid volume of 50 mL under similar conditions, except that vanillin and KI concentrations were increased to 100 mM and 110–200 mM, respectively and 0.25 mg mL^−1^ laccase was added a second time after 5 h. Foaming was prevented by the addition of 50 µL silicon antifoam (Sigma-Aldrich). Precipitated products were separated by sedimentation for 30 min in 50 mL plastic tubes and the insoluble material was washed 2× with 20 mL ddH_2_O. The maximal solubility values for vanillin and 5-iodovanillin in different solvents were approximated by cumulative addition of the compounds to 2–50 mL solution in 5 mg steps. The maximal solubility of vanillin and 5-iodovanillin under biotransformation conditions (McIlvain buffer with 5% v/v ethanol, pH 5.0) was ≈115 mM and <0.7 mM, respectively. The maximal solubility values in ethyl acetate were 1.6 M for vanillin and 73 mM (20 g L^−1^) for 5-iodovanillin. Degradation of 5-iodovanillin by laccase was analyzed by incubating 2 mM of the commercial compound (added as 20-fold concentrated stock solution in DMSO) in the presence of 0.25 mg mL^−1^ *T. versicolor* enzyme in McIlvain buffer at pH 5.0 and room temperature for 3 days under light-protected conditions.

### HPLC-MS analysis

Ethyl acetate extracts of biotransformations and control reactions were analyzed with an Agilent 1100 series HPLC device equipped with a G1315A UV/DAD detector (Agilent Technologies Inc., Santa Clara, CA, USA) which was coupled to a Bruker Daltonics Esquire HCT mass spectrometer (Bruker Daltonics Inc., Billerica, MA, USA). A Gemini C18 reversed phase column (110A, 250×4.6 mm, 5 micron, Phenomenex, Torrance, CA, USA) was used for separation of phenolic compounds. The mobile phase consisted of a gradient of 60–100% acetonitrile in ddH_2_O within 10 min at a flow rate of 0.8 mL min^−1^ and the sample injection volume was 10 µL. All eluents were supplemented with 0.1% v/v formic acid. Retention times and mass spectra of educts and commercially available 5-iodovanillin were determined with 1 mM and 2 mM solutions of the respective compounds in ethyl acetate. Conversion of phenolic compounds was quantified by calculating the relative reduction of UV peak areas (275 nm) in enzymatic reactions compared to enzyme-free controls. Concentrations of vanillin and iodovanillin at different time points were quantified by linear regression from HPLC-UV standard curves determined with a concentration series of commercially available pure compounds (0.1 to 2.5 mM). The identity of product HPLC-UV peaks in ethyl acetate extracts of biotransformations was determined by overlay with extracted ion chromatograms for theoretical *m/z* values of possible products. Purity and yield of identified products were estimated from relative UV peak areas.

### Biotests

The antifungal activity of commercial 5-iodovanillin and biocatalytically produced iodovanillin/iodo-ethyl vanillin was tested on agar plates (Ø 90 mm). Iodinated compounds were dissolved to a concentration of 0.4 M in DMSO (stock solution). The non-iodinated reference compounds (educts) vanillin and ethyl vanillin were dissolved to a concentration of 0.1 M in ethanol. The compounds were added to 2% w/v malt extract agar after autoclaving to final concentrations of 1, 2 and 4 mM, respectively. Reference plates for determination of relative growth inhibition contained similar amounts of DMSO or ethanol as added with stock solutions, but no phenolic compounds. Agar plates were inoculated in the center with a mycelium agar disc (9 mm diameter) taken from the submargins of 14-day old cultures of the basidiomycetes *Oligoporus placenta* (Empa strain No. 45), *Gloephyllum trabeum* (Empa strain No. 100), *Coniophora puteana* (Empa strain No. 62), *Trametes versicolor* (Empa strain No. 159) and the ascomycete *Aureobasidium pullulans* (Empa strain No. 316). Plates were incubated at 22°C and 70% relative humidity. The radial fungal growth was estimated by measuring the colony diameter, and the growth inhibition was expressed as percentage reduction of this value after similar incubation times. Furthermore, so called drop tests for secreted enzymes were performed according to Stalpers *et al.* [13]. Laccase and peroxidase activities were analyzed with α-naphthol (0.1 M in ethanol) and pyrogallol (0.4% v/v H_2_O_2_ and 1% v/v pyrogallol in ddH_2_O) solutions, respectively. A purple color occurs when laccase is present and a yellow-brown color is formed by secreted peroxidase. All reactions were performed on actively growing marginal hyphae (mycelium at the edge of the colony) and read after 24 hours. The ability of the compounds to kill fungi (biocidal effect) during the time of incubation was evaluated by aseptically transferring three mycelia discs (Ø 5 mm) from test plates with phenolic compounds to fresh malt extract agar plates without supplements. The absence of regrowth of hyphae around the discs was considered as biocidal effect.

For the statistical analysis of growth inhibition, colony diameter data of 6 replicate plates per compound/control were arcsine transformed prior to statistical analysis (ANOVA) and back transformed to numerical values for visualization. Means were separated using independent t-test and Tukey's HSD (honestly significant difference) test at significance levels with *P* values of <0.05 and <0.001. The statistical package used for all analyses was SPSS (version 17.0; SPSS Inc., Chicago, IL).

## Results

### Kinetics of enzymatic iodide oxidation

Laccases are capable of oxidizing colorless iodide (I^−^) in aqueous solutions to iodine (I_2_), in turn brownish-colored triiodide (I_3_^−^) is formed from I^−^ and I_2_ which can be measured at 353 nm. In order to find the optimal conditions for a potential bio-iodination of phenolic compounds we determined the pH optimum and the dependency of iodide oxidation rates on substrate concentration for a commercially available laccase preparation from *Trametes versicolor*. The pH optimum for iodide oxidation by *T. versicolor* laccase without the addition of mediator or secondary substrate was 2.6. The addition of two known redox mediators, ABTS and acetosyringone (a highly efficient natural laccase mediator, [14]), in a 1∶5000 molar ratio to iodide (10 µM) increased the pH optimum for iodide oxidation to 5.0 and 5.2, respectively. In the presence of low concentrations of the potential iodination substrate vanillin the pH optimum was also increased to values of 4.2 (10 µM vanillin) to 4.6 (100 µM vanillin). Vanillin is known to be oxidized by laccases and was proposed to act as (weak) mediator [14]. Based on these results, a pH of 5.0 was generally used for biotransformation experiments.

**Figure 1:**
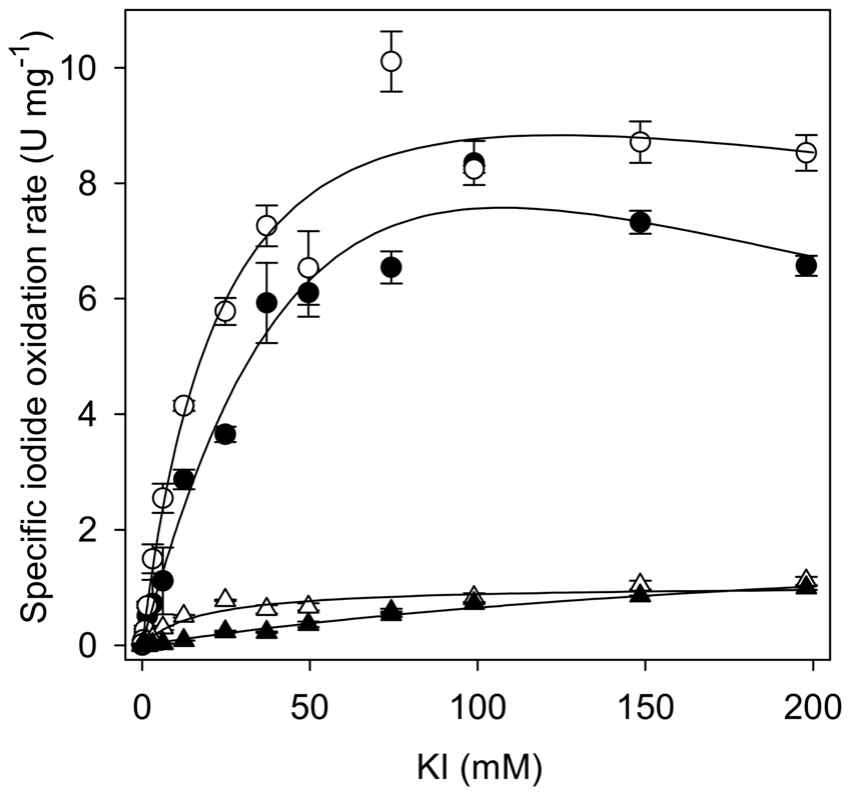
Dependency of specific iodide oxidation rates on iodide concentration for *T. versicolor* laccase. Reaction conditions: Citrate-phosphate buffer pH 5.0, room temperature, 0.01 or 0.1 mg mL^−1^ enzyme. Closed triangles: without organic substrate, open triangles: with vanillin (100 µM), filled circles: with mediator ABTS (10 µM), open circles: with mediator acetosyringone (10 µM). Average values and standard deviations of n = 3 replicate assays per concentration. Black lines: best fit (least squares) of Michaelis-Menten models (incl. substrate inhibition).

Specific iodide oxidation rates determined for *T. versicolor* laccase showed a typical Michaelis-Menten like dependency on substrate concentration (Figure 1). The addition of low concentrations of the mediators ABTS and acetosyringone, but not of vanillin increased maximal specific iodide oxidation rates by a factor of 6 to 10 (Table 1). The presence of either ABTS, acetosyringone or vanillin significantly decreased the apparent *K*_M_ of iodide oxidation (Table 1). Considering a molecular weight of 70 kDa for *T. versicolor* laccase [15], the enzyme concentration in experiments with the best-performing mediator ABTS was 430 nM. The maximal iodide oxidation rate achieved under these conditions was 2505±114 µM min^−1^ (n = 3) at 100 mM KI. Due to the relatively high apparent *K*_M_ value, the iodide oxidation rate at a concentration of 1 mM KI was 37-fold lower (67±35 µM min^−1^, n = 3). In the light of these results, 0.1 mM ABTS and a potassium iodide concentration of at least 50 mM were used in biotransformations in order to achieve reasonably high iodide oxidation rates.

**Table 1:** Apparent kinetic parameters for iodide oxidation by *T. versicolor* laccase approximated by non-linear regression.

Condition	*K*_M_ [mM]	*K*_i_ [mM]	*V*_max_ [U mg^−1^]
KI	254 (±38)	-	2.3 (±0.23)
KI + 100 µM vanillin	18 (±3.4)	-	1.04 (±0.05)
KI + 10 µM ABTS	95 (±35)	122 (±56)	21 (±5.8)
KI + 10 µM acetosyringone	26 (±5.5)	580 (±273)	13 (±1.3)

### Laccase-catalyzed iodination of vanillin and related compounds

**Figure 2:**
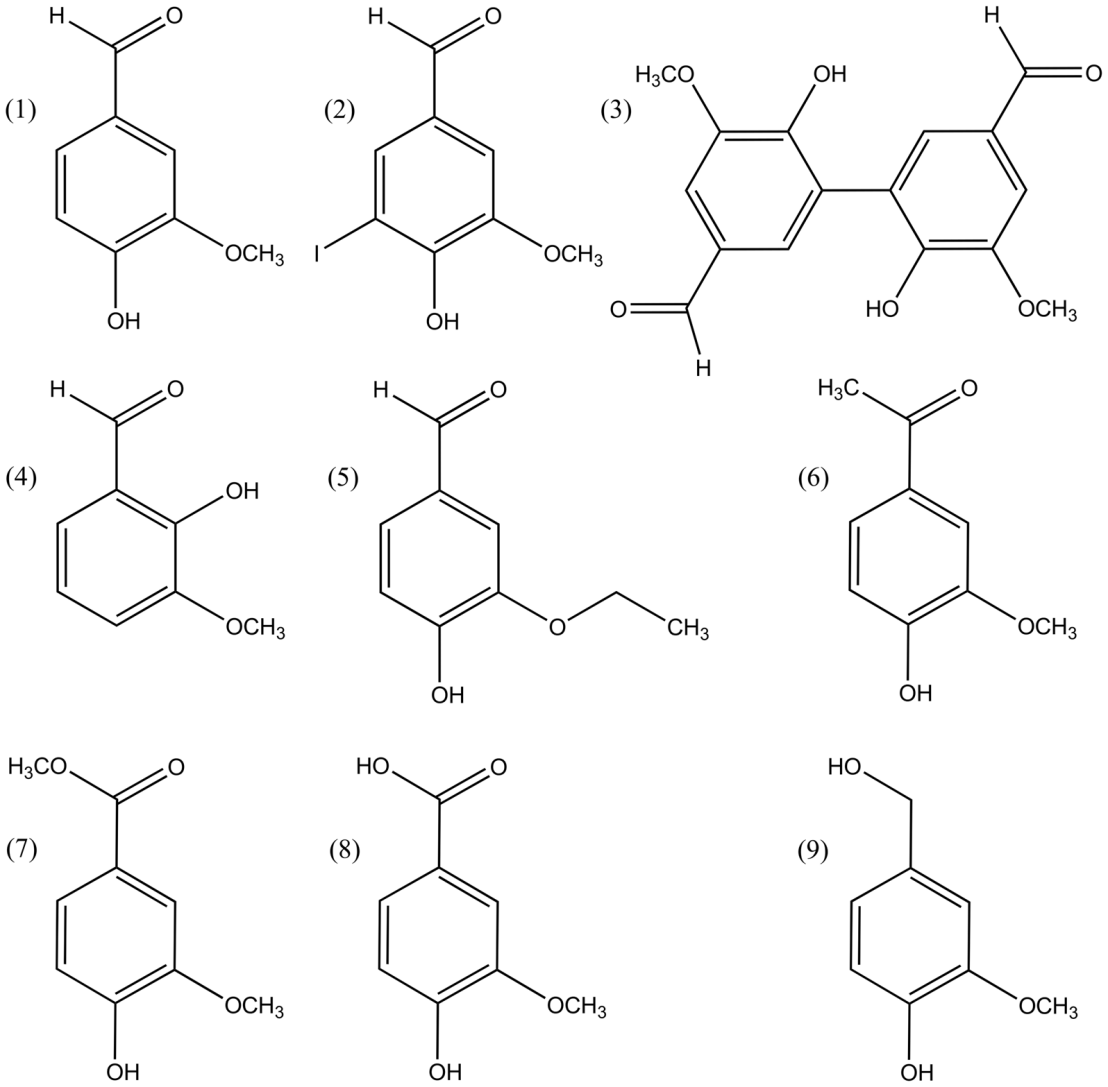
Educts (1 and 4–9) and selected assumed products (2, 3) of laccase-iodide biotransformations. Molecular masses in g mol^−1^ are given in brackets. (1) Vanillin [152.15], (2) 5-iodovanillin [278.05], (3) divanillin [302.28], (4) *o*-vanillin (152.15), (5) ethyl vanillin [166.18], (6) acetovanillone [166.18], (7) methyl vanillate [182.17], (8) vanillic acid [168.15], (9) vanillyl alcohol [154.16].

Indirect evidence of previous studies indicated that laccase catalyzed iodide oxidation is involved in the formation of iodinated organic compounds in soil organic matter and in the lignin fraction of wood [9], [10]. Therefore, we tested whether iodination of simple phenolic compounds occurs in the presence of laccase and unreactive potassium iodide. Vanillin (compound 1, Figure 2) was selected as model educt because it has a similar structure as the dominant coniferyl alcohol monomers of softwood lignin [16] and is itself a laccase substrate [14]. Furthermore, vanillin can be iodinated relatively easily under alkaline conditions using I_2_/KI solutions [17].

**Figure 3:**
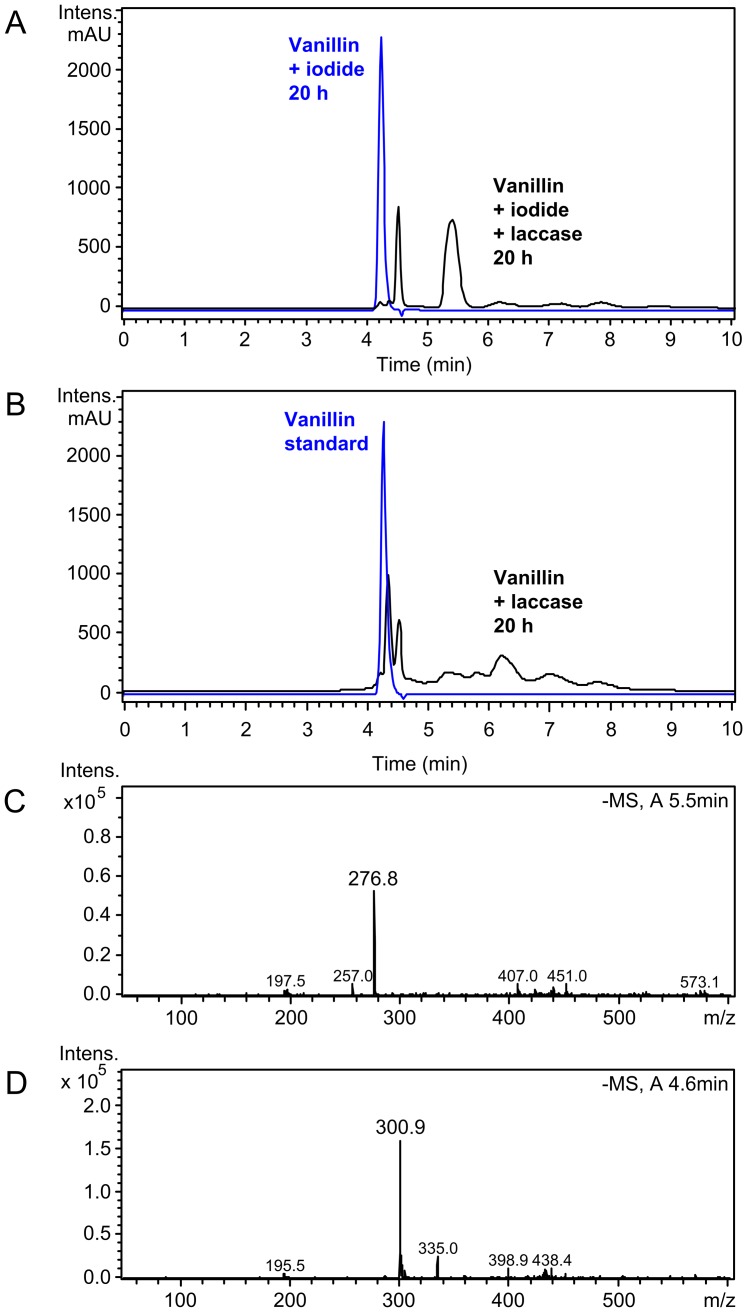
HPLC-MS analysis of vanillin biotransformations. (A) Overlay of UV chromatograms (absorbance at 275 nm) of ethyl acetate extracts of a laccase-iodide-vanillin biotransformation (black trace) and a laccase-free control reaction (blue trace). (B) Overlay of UV chromatograms (275 nm) of ethyl acetate extracts of a laccase-vanillin biotransformation (black trace) and a vanillin standard (1 mM, in ethyl acetate). (C) Mass spectrum for the major product peak of A with a retention time of 5.2–5.6 min. (D) Mass spectrum for the first product peak of A with a retention time of 4.4–4.6 min.

Biotransformation reactions containing vanillin, iodide and laccase were incubated under oxic conditions for 20 h and reaction products were extracted with ethyl acetate. Comparison of HPLC-UV chromatograms of these reactions with laccase-free controls revealed complete conversion and formation of several products with increased retention times (Figure 3A). The HPLC-UV chromatogram and mass spectrum of the single visible peak of the laccase-free control reaction matched that of a vanillin standard (Figures 3A and 3B), confirming that iodide did not react with vanillin without catalysis. In the laccase-iodide-vanillin biotransformation a major product peak occurred at a retention time of 5.5 min (Figure 3A, black trace). The dominant mass of this peak was *m/z* = 277 (neg. mode), which corresponds to the molecular mass of 5-iodovanillin (2) with abstracted hydrogen atom ([M-H]^−^, Figures 2 and 3C). Retention time and mass spectrum of commercial 5-iodovanillin dissolved in ethyl acetate were similar to this product peak (Figure S1). The dominant mass of a second prominent product peak at a retention time of 4.6 min was *m/z* = 301 (neg. mode) which corresponds to the theoretical molecular mass of divanillin (3) with abstracted hydrogen atom ([2M_Vanillin_-3H]^−^, Figures 2 and 3D). Although MS analysis does not allow to determine the exact structure of the assumed dimer, it is likely that a 5,5′ C-C coupling occurred as shown for compound (3) in Figure 2. Others have shown that a 5,5′ dimer was the predominant product of laccase catalyzed oxidation of vanillyl alcohol at pH 4.5 to 7.5 [18]. Oxidative dimerization of vanillin by horseradish peroxidase at pH 4 also lead to the 5,5′ product [19]. Minor product peaks in Figure 3A with higher retention times than those of iodovanillin presumably represented multimeric coupling products of vanillin, either iodinated or non-iodinated. However, no dominant masses corresponding to specific (iodo)vanillin multimers could be identified. The addition of 100 µM ABTS as redox mediator to laccase-iodide-vanillin biotransformations (1∶40 molar ratio to vanillin, 1∶500 molar ratio to iodide) increased the fraction of iodovanillin in the product mix from 59 to 90% relative peak area, while the fraction of the assumed most abundant byproduct divanillin decreased (Table 2).

**Table 2:** Laccase-catalyzed iodination of different mono-methoxy-substituted phenolic compounds (structures: see Figure 2).

Educt	% Conversion^a^	Assumed products^b^ (*m/z*, neg. MS)	% Peak area^c^
Vanillin (1)	97±0.3	iodovanillin (277)	90±0.1
		divanillin (301)	2.7±1.0
Vanillin w/o mediator	99±0.03	iodovanillin (277)	59±0.5
		divanillin (301)	37±0.6
*o*-vanillin (4)	73±3	iodo-*o*-vanillin (277)	17.4±1.7
		di-*o*-vanillin (301)	7.0±1.1
		tri-*o*-vanillin (451)	7.8±1.5
Ethyl vanillin (5)	99±0.2	iodo-ethyl vanillin (291)	82±5.7
		di-(ethyl vanillin) (329)	8.4±2.3
Acetovanillone (6)	99±0.2	iodo-acetovanillone (291)	83±1.2
		di-acetovanillone (329)	12±1.0
Methyl vanillate (7)	97±0.2	iodo-methyl vanillate (307)	87±0.9
		di-(methyl vanillate) (361)	12±1.0
Vanillic acid (8)	97±1.3	iodo-vanillic acid (293)	19±4.5
Vanillyl alcohol (9)	96±0.7	iodovanillin (277)	10±0.8
		iodo-vanillic acid (293)	17±2.6

a Reaction conditions: 4 mM educt, 50 mM potassium iodide, 100 µM ABTS and 0.25 mg mL^−1^ *T. versicolor* laccase, % conversion was calculated as the relative decrease in educt peak area compared to enzyme-free control reactions.

b Reaction batches were extracted with ethyl acetate and educts and products were analyzed by HPLC-UV/MS, molecular mass of iodide atom: 126.9 g mol^−1^, for molecular masses of educts see Figure 2.

c Relative peak area of products, average values and standard deviations of n = 3 replicate experiments.

Mono-iodinated products could also be detected in laccase-iodide biotransformations of other educts with related chemical structures (Figure 2, Table 2). High yields of iodo-compounds were obtained for the educts ethyl vanillin, acetovanillone and methyl vanillate, while *o*-vanillin and vanillic acid were transformed to the iodinated forms less efficiently (Table 2). Vanillyl alcohol was also completely converted, but only iodovanillin and iodo-vanillic acid could be identified as products, indicating laccase catalyzed oxidation of the aliphatic hydroxyl group before or after iodination. In most cases, assumed dimers of the educt were also formed, but usually in lower amounts than the iodinated products (Table 2).

In biotransformation reactions containing only laccase and vanillin, the educt was converted to a complex mixture of compounds with overlapping, extended retention times (Figure 3D). The second product peak with a retention time of 4.6 min was also assumed to represent divanillin due to the corresponding mass spectrum (*m/z* = 301, neg. mode). Thus, the presence of the reducing agent iodide apparently suppressed the formation of higher molecular weight coupling products (vanillin oligomers). The formation of a mixture of different oligomer byproducts is a recurring problem in laccase reactions [4]. Others have also shown that the ratio of different coupling products in laccase biotransformations can be strongly influenced by reactions conditions, e.g., the type of organic solvent [20].

### Time course of enzymatic vanillin iodination

**Figure 4:**
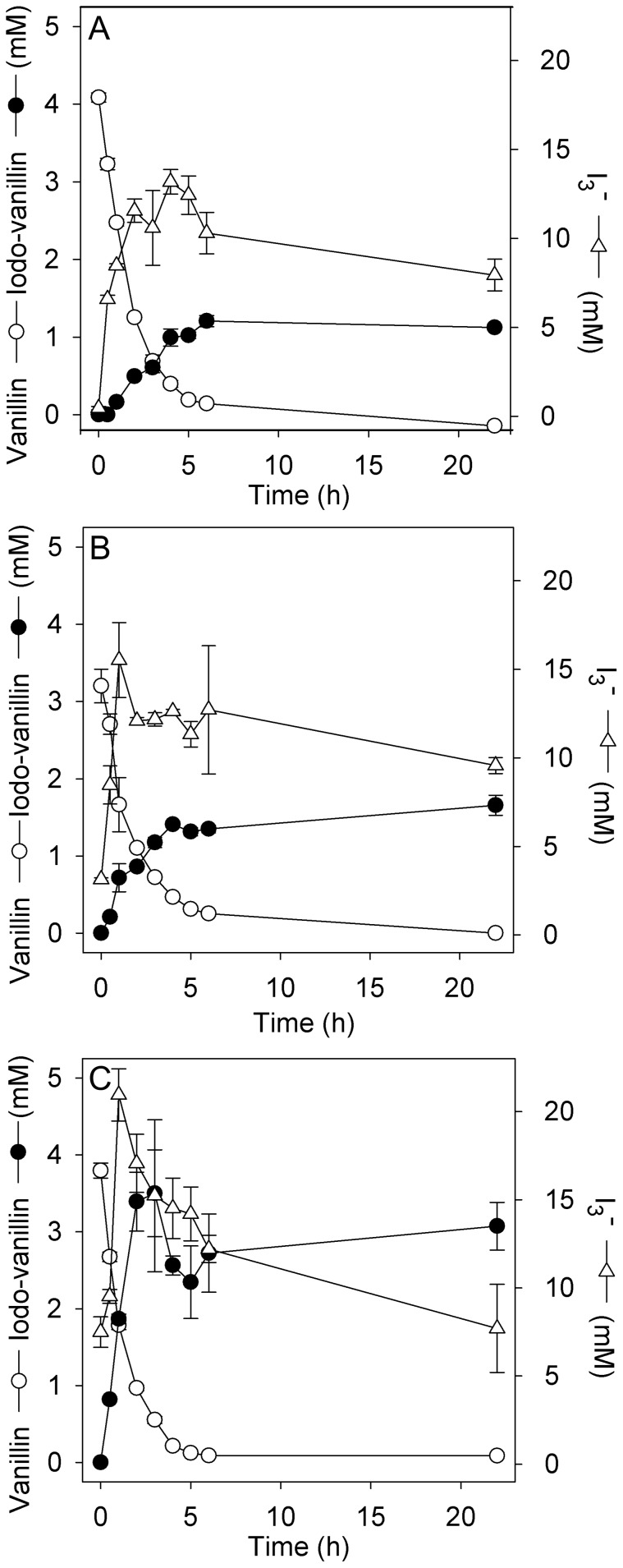
Time course of laccase catalyzed iodination of vanillin. Biotransformations (A) without mediator, (B) with mediator acetosyringone (100 µM), (C) with mediator ABTS (100 µM). Reaction conditions: 4 mM vanillin, 50 mM KI and 0.25 mg mL^−1^ *T. versicolor* laccase in McIlvain buffer (pH 5.0), open flasks (V_liquid_ = 50 mL) shaken at 150 rpm at room temperature. Average values and standard deviations (error bars) of n = 3 replicate experiments.

The time course of laccase catalyzed iodination of vanillin was determined in triplicate shake flask experiments with and without mediators. Vanillin (4 mM) was completely converted within 6 h without mediators (Figure 4A), with 100 µM acetosyringone (Figure 4B) or with 100 µM ABTS (Figure 4C), but the extent to which it was transformed to iodovanillin differed between the experiments.

In all enzymatic reactions excess triiodide was produced which was not fully consumed until the end of the experiment (Figure 4). In agreement with the enhancing effect of mediators on iodide oxidation (Figure 1), accumulation rates and maximal levels of triiodide were higher in acetosyringone and ABTS-enhanced biotransformations (Figures 4B and 4C). In accordance with this observation, the rate of iodovanillin formation and its final yield were also increased in the presence of mediators (Figure 4). The molar ratio of iodovanillin product to consumed vanillin was highest in ABTS-supplemented reactions. Although vanillin was almost completely converted within 6 h under all tested conditions, a reaction time of 20 h was chosen for preparative, up-scaled biotransformations and for biotransformations of other phenolic compounds for simplicity reasons.

In all biotransformations the rate of vanillin conversion continuously decreased (Figure 4), indicating loss of laccase activity during the experiments. We analyzed laccase activity at the beginning and end of biotransformations with the standard ABTS assay. While the initial enzyme activity (4 U mL^−1^) in laccase-vanillin biotransformations was reduced by only 34% after 20 h of incubation, laccase was completely inactivated after the same time in laccase-iodide-vanillin biotransformations (>99% reduction of initially added activity). Presumably, the laccase protein structure was negatively affected by reaction with oxidized iodine species, (e.g. iodination of tyrosine and histidine residues, [21]).

### Preparative bio-iodination

It was possible to scale up laccase catalyzed, ABTS-mediated iodination of 4 mM vanillin and ethyl vanillin from 2 mL to 1 L scale in aerated Erlenmeyer flasks. Products could be extracted with 1∶3 v/v ethyl acetate and recovered in solid form in a rotary evaporator. HPLC-UV analysis of re-dissolved solid products revealed product purities of 93% and 94% for iodovanillin and iodo-ethyl vanillin, respectively (relative peak areas). Retention times and mass spectra of the major impurity peaks corresponded to the respective assumed dimers (see also Table 2). Laccase-catalyzed iodination could also be carried out at a 25-fold higher educt concentration (100 mM) which is close to the maximal solubility of vanillin under the chosen biotransformation conditions (115 mM in McIlvain buffer pH 5.0 with 5% v/v ethanol). The solubility of iodovanillin in the same buffer was found to be below 0.7 mM, therefore the product of laccase-catalyzed vanillin iodination precipitated and could be isolated by simple sedimentation or centrifugation. After washing with double distilled water and re-dissolving in ethyl acetate, the purity of this enzymatically synthesized iodovanillin was determined to be 74% by HPLC-UV. The preparation contained 15% residual vanillin educt and 11% assumed divanillin byproduct (relative HPLC-UV peak areas). When using high vanillin educt concentrations, the molar excess of iodide could be reduced to values as low as 1.1: 1. The volumetric yield of precipitated iodovanillin after 20 h of incubation under these conditions was 9.4±0.6 g L^−1^ (34 mM, n = 3 replicate biotransformations). HPLC-UV analysis of ethyl acetate extracts of the remaining liquid phase showed that only about 50% of the initial 100 mM vanillin were converted, most likely due to inactivation of laccase by reactive iodine species. Taking incomplete educt conversion into account, the vanillin to iodovanillin conversion rate in these biotransformations was 68%. While it was possible to reduce the KI to vanillin molar ratio from 12.5 to 1.1 at 100 mM educt concentration without reducing iodovanillin yields considerably, this was not possible in biotransformations with low vanillin concentrations (4 mM). Here, any reduction of the KI to vanillin molar ratio to values below 2 (8 mM KI) strongly decreased iodovanillin yields (relative peak areas <10%), presumably because iodide oxidation rates became too slow (see also Figure 1). In chemical control reactions concentrated I_2_/KI (Lugol's solution) was added to 4 mM vanillin in McIlvain buffer in different molar ratios. After 20 h incubation at room temperature iodovanillin was also formed. The relative UV peak area of iodovanillin product to vanillin educt decreased from 82% at a total iodine to vanillin molar ratio of 5∶1 to 9% when a molar ratio of 1.5∶1 was used, confirming the low atom efficiency of conventional iodination.

### Antifungal activity of bio-iodinated phenolic compounds

In a previous study we found that treatment of spruce wood with laccase and iodide prevented growth of various fungal species when inoculated onto the surface. The antifungal effect resisted rigorous washing with water, indicating modification of polymeric components of the treated wood. In order to study whether iodinated phenolic materials (i.e. lignin) contributed to this effect, we analyzed the antifungal activity of iodovanillin and iodo-ethyl vanillin which had been synthesized by the use of the biocatalyst laccase. Both compounds significantly reduced the growth of four different basidiomycete species at all tested concentrations (Table 3). The effectiveness of commercial, chemically synthesized 5-iodovanillin was similar to that of enzymatically synthesized material (Table 3). No inhibitory effect was found for the ascomycete fungus *A. pullulans*. Almost complete growth inhibition by iodovanillin and iodo-ethyl vanillin was observed for *T. versicolor*, a basidiomycete species that causes white rot decay of wood (Table 3). Here, also a partial biocidal effect was observed because no actively growing mycelia were recovered for one out of three and two out of three re-inoculated discs taken from plates with 2 and 4 mM iodovanillin, respectively.

**Table 3:** Relative growth inhibition (%) of filamentous fungi by (bio-)iodinated phenolic compounds and corresponding non-iodinated compounds.

Fungal species	Vanillin^a^^,^ b			Ethyl vanillin^a^^,^ b			5-Iodovanillin (commercial)^a^^,^ b			Iodovanillin (from biotransformation)^a^^,^ b			Iodo-ethyl vanillin (from biotransformation)^a^^,^ b		
	4 mM	2 mM	1 mM	4 mM	2 mM	1 mM	4 mM	2 mM	1 mM	4 mM	2 mM	1 mM	4 mM	2 mM	1 mM
*O. placenta*	55.28*	11.76	0.00	0.00	0.00	0.00	9.06*	22.51**	17.73**	16.78**	27.61**	8.30*	20.47**	33.64**	18.64*
	±18.9	±8.5	±0.0	±2.7	±0.7	±0.0	±3.1	±1.1	±0.4	±1.5	±1.2	±0.5	±1.0	±0.4	±0.4
*G. trabeum*	51.36*	13.38*	2.19	32.59**	23.90*	15.82*	38.14**	37.28**	40.88**	49.45**	50.22**	39.34*	70.07**	70.18*	49.45**
	±10.4	±6.1	±1.0	±0.4	±2.0	±2.5	±2.3	±1.0	±0.4	±2.0	±5.0	±7.1	±1.8	±6.0	±3.0
*T. versicolor*	42.00*	18.75*	6.59	11.40*	29.93*	10.16*	82.89**	82.04**	78.52**	95.83**	85.81**	72.52**	99.67**	97.67**	95.84**
	±9.3	±4.0	±4.2	±3.3	±5.1	±1.1	±4.7	±2.9	±2.8	±1.5	±3.4	±6.2	±0.3	±0.3	±0.7
*C. puteana*	61.48*	24.17*	18.24	31.18	21.08	0.00	56.45*	65.11**	66.41**	71.24*	67.92**	67.05*	74.19*	53.63**	52.78*
	±6.1	±6.8	±31.6	±21.2	±18.3	±5.3	±2.8	±8.9	±6.3	±14.7	±3.9	±11.9	±7.7	±3.9	±19.3
*A. pullulans*	17.93	8.52	0.00	–	–	–	1.51	7.31*	1.36	–	–	–	–	–	–
	±11.5	±14.8	±0.0				±2.4	±1.9	±1.4						

a Growth inhibition (% reduction of growth radius) on additive-containing malt agar plates in comparison to phenolic compound-free control plates, average values and standard deviations of n = 6 replicate plates.

b Significant growth inhibition compared to control is marked by * = *P*<0.05 and ** = *P*<0.001 (independent T-test).

**Figure 5:**
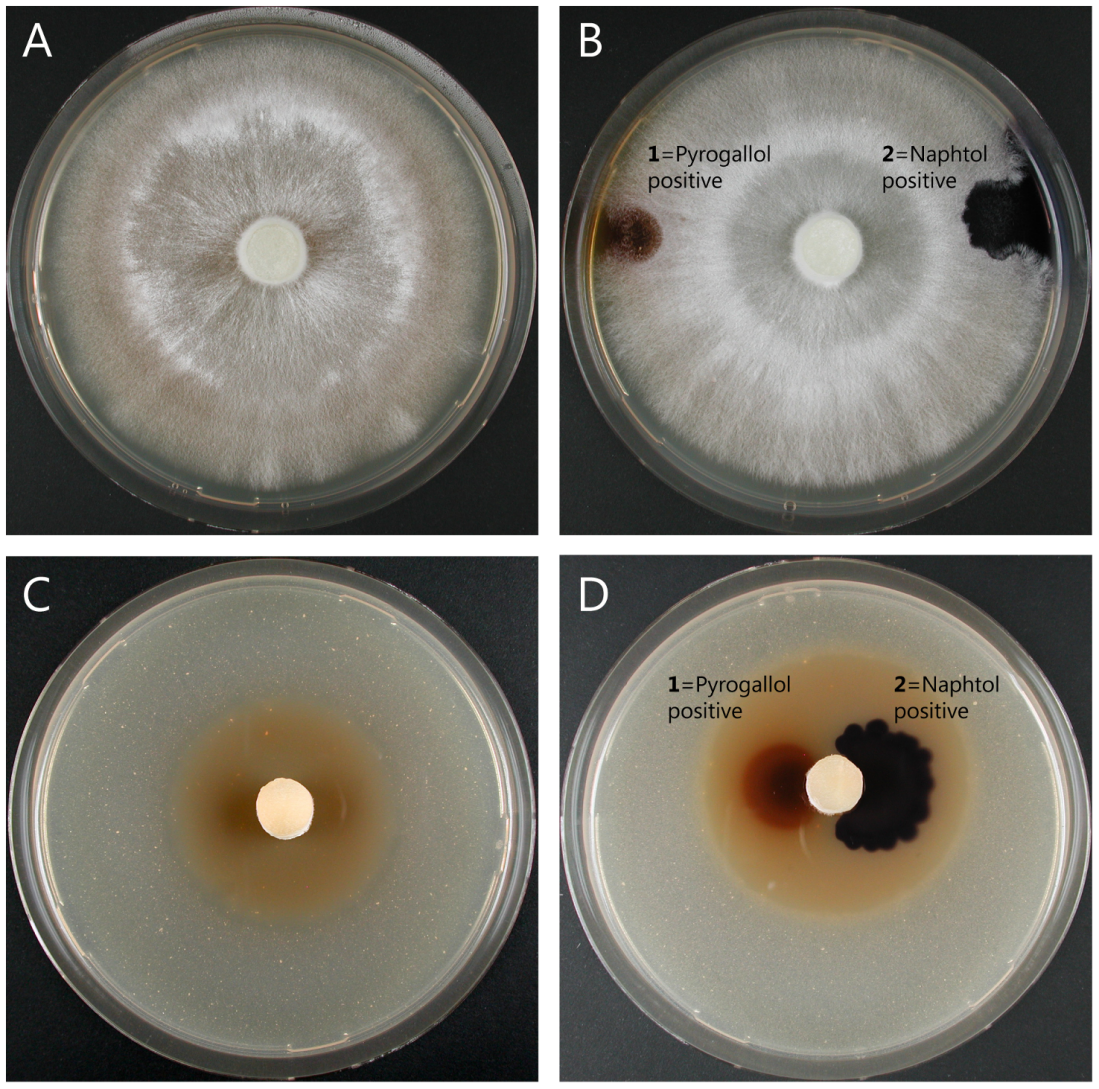
Growth inhibitory effect of bio-iodinated ethyl vanillin on *T. versicolor* and qualitative analysis of secreted oxidative enzymes. (A) Malt agar plate with 2 mM ethyl vanillin after 14 d of incubation, (B) a similar plate after pyrogallol and naphthol drop test for detection of secreted peroxidase (1) and laccase (2), (C) malt agar plate with 2 mM iodo-ethyl vanillin after 14 d of incubation, note the brownish halo around the inoculated disc which indicates iodine release, (D) a similar plate after pyrogallol and naphthol drop test.

No or only marginal growth inhibitory effects were observed for the educts vanillin and ethyl vanillin at a concentration of 1 mM. At higher concentrations these compounds significantly reduced growth of some of the tested species, but to a significantly lower extent than the respective iodinated compounds (Table 3). An example for the growth difference on malt agar plates containing similar concentrations of educt (ethyl vanillin) and product (iodo-ethyl vanillin) is shown in Figure 5 for *T. versicolor*. Interestingly, a light brown halo formed around the inoculated mycelia disc on iodo-ethyl vanillin (Figure 5C) and iodovanillin plates (not shown). This could be the result of iodine release due to decay of the iodinated phenols by secreted oxidative enzymes. Therefore, we tested whether *T. versicolor* constitutively secreted such enzymes on malt agar test plates. Both peroxidases (pyrogallol drop test) and laccases (naphthol drop test) were detected on control plates without phenolic compounds and on plates containing either ethyl vanillin or iodo-ethyl vanillin (Figures 5B and 5D, respectively). Next we tested whether commercial *T. versicolor* laccase can also catalyze the backward reaction of vanillin iodination. When 5-iodovanillin was incubated with laccase for 3 days, the corresponding HPLC-UV peak was reduced by 57.2±1.2% (n = 3 replicate experiments) compared to an enzyme-free control and several product peaks with retention times of 6 to 8 min appeared in the chromatogram. At the same time a brownish color was formed which was absent from the control. Judged from the absorbance at 353 nm measured in ethyl acetate extracts (to circumvent the background absorbance of laccase), 0.15±0.004 mM triiodide was formed. This indicates that reactive iodine species can be released from iodinated phenols by the action of *T. versicolor* laccase.

The secretion of peroxidases and laccases on malt agar test plates was also analyzed for the other fungal species which were less sensitive to iodovanillin and iodo-ethyl vanillin (Table 3). The pyrogallol and naphthol drop tests were negative for all other basidiomycete (brown rot) and ascomycete fungi grown on malt agar plates. This is in agreement with the exclusive appearance of brown halos around inoculated mycelia on *T. versicolor* plates with iodovanillin and iodo-ethyl vanillin and explains the significantly higher susceptibility of this species to the iodo-compounds.

## Discussion

In this study we demonstrated that iodinated compounds with antifungal activity can be synthesized by a simple biocatalytical process from lignin-related substituted phenols. Biocatalytic approaches are promising for reducing the environmental impact of chemical synthesis processes and products [22]. Due to the intrinsic characteristics of most enzymatic reactions, key demands of green chemistry can be met such as chemo-selectivity, high rate accelerations (catalysis), biocompatibility, high atom efficiency, reduction of the use of organic solvents, non-hazardous synthesis procedures and energy efficiency by avoiding high temperatures and pressures [23], [24].

Biocatalytic strategies have also been suggested as alternative synthetic approach to the halogenation of organic molecules [25]. Aromatic iodo-compounds are widely applied in the synthesis of complex organic molecules, however their conventional preparation using elemental iodine is inefficient compared to chlorination and bromination and requires activation for effective reactions such as electrophilic substitution [26]. Laccase catalyzed iodination of phenolic compounds as described in this study fulfills key criteria of a green chemistry process [23]. The reaction can be performed under ambient pressure and temperature in aqueous solution, using chemically stable, non-reactive iodide salts in low molar excess as starting material.

The novel enzyme initiated iodination reaction most likely relies on the laccase catalyzed iodide-to-iodine oxidation. However, the system is rather complex and a detailed reaction mechanism cannot be inferred easily. The reaction batch contains a multicopper enzyme, substrate I (a methoxy-substituted phenolic compound such as vanillin), substrate II (iodide), in some cases substrate III (a mediator such as ABTS) and a co-substrate (oxygen). During the course of reaction it is clear from colorimetric measurements that I^−^ is oxidized to I_2_ which immediately reacts with remaining I^−^ to I_3_^−^. Simultaneously vanillin (or another, similar compound) probably is oxidized to the phenoxy radical that undergoes radical coupling [18], [27], as indicated by formation of a byproduct with the *m/z* value of divanillin. In the case of biotransformations with mediators, ABTS (or acetosyringone) is most likely oxidized to the stable radical cation ABTS^•+^ (or the stable phenoxy radical of acetosyringone) [28], [29]. These radicals then oxidize additional iodide ions and/or vanillin and are in turn back-reduced to the original state (closing the mediator cycle). In parallel with these processes, iodovanillin is formed, either by interaction of reactive iodine species (I_2_, I_3_^−^) with unmodified vanillin, with vanillin phenoxy radicals or even with phenolate anions. Presumably, the oxidized iodine species generated by laccase catalysis act as electrophiles for the electron-dense aromatic rings of vanillin and related compounds, similar to the mechanism suggested for conventional iodination methods [26]. Elucidation of the exact sequence of events and the molecular mechanisms governing the interactions of laccase, iodide, mediators and phenolic compounds requires extensive further investigations and is without the scope of this study.

The addition of the redox mediator ABTS in catalytic concentrations increased both, iodide oxidation rates and final iodovanillin yields compared to biotransformations with only vanillin and iodide, although the velocity of vanillin conversion was similar in both cases (Figure 4). Effective redox mediators cycle between the oxidized (radical) and the reduced state without undergoing futile, dead-end side reactions. Experimental evidence indicates that vanillin is not an effective redox mediator for laccases, although it can be oxidized by this enzyme [14]. In ABTS-free bio-iodination reactions a significant part of the oxidized vanillin was found to be converted to a byproduct which is assumed to be divanillin (Table 2), presumably by radical coupling. Apparently, the vanillin radicals arising from laccase-catalyzed oxidation did not efficiently react with iodide, but rather oligomerized, which is in agreement with the limited effect of vanillin on iodide oxidation rates (Figure 1, Table 1). A possible explanation for the increased iodovanillin yield in the presence of ABTS is that reduced ABTS had a higher affinity for the active site of laccase than vanillin, thereby diminishing the formation of oligomerization-prone vanillin radicals. Acetosyringone, which is known to be a good redox mediator for laccases [14] did not increase the relative iodovanillin yield to a similar extent as ABTS (Figure 4), although it was at least as effective in increasing iodide oxidation rates (Figure 1). ABTS and acetosyringone have been shown to have a similar apparent *K*_M_ value in mediated reactions catalyzed by a *Trametes* sp. laccase [30], therefore, it is unlikely that acetosyringone is less effective than reduced ABTS in competing with vanillin for the active site. However, it could be that due to unknown reasons ABTS radicals preferentially oxidize iodide, while acetosyringone radicals react both with iodide and vanillin, which leads to the (indirect) formation of oligomerization-prone vanillin radicals.

The commercial *T. versicolor* preparation used for the iodination experiments exhibited a very high specific activity for iodide oxidation, in particular in the presence of redox mediators. Previously, it was shown that recombinant fungal laccases from *Myceliopthora thermophila*, *Polyporus pinsitus*, *Coprinus cinereus*, and *Rhizoctonia solani* are capable of iodide oxidation [3], [7]. *P. pinsitus* laccase was the best-performing enzyme, exhibiting a maximal iodide oxidation rate of 6 µM min^−1^ with 10 µM methyl syringate as mediator. Although the enzyme concentrations used in our study were in the same range as in the study of Kulys *et al.* [7], we found a 10-fold higher iodide oxidation rate for the *T. versicolor* laccase at a similar KI concentration (1 mM). This indicates that *T. versicolor* laccase is exceptionally well suited for iodide oxidation, which may be due to the high redox potential of this enzyme (E^0^ = 0.79 V) [31]. Enzymes with lower redox potential such as *M. thermophila* laccase (E^0^ = of 0.5 V) exhibit considerably lower iodide oxidation rates [7].

Due to the tolerance of *T. versicolor* laccase to both high vanillin and potassium iodide concentrations, it was possible to achieve high volumetric yields of the iodinated compound and to recover the reaction product by simple sedimentation or centrifugation. It is likely that yields can be further increased by optimizing laccase and mediator concentrations, e.g. with a neural network approach [32]. Fed-batch type biotransformations with continuous addition of phenolic educts, iodide and laccase may also facilitate even higher volumetric yields. A drawback of laccase-iodide biotransformation is the strong loss of enzyme activity which is most likely due to the attack of the laccase polypeptide by reactive iodine species. Laccase inactivation in iodination reactions might be reduced by using immobilized biocatalysts. Enzyme immobilization often results in enhanced stability [33], e.g., covalent attachment of laccase to silica microspheres resulted in a strongly improved resistance to heat denaturation [34].

Due to the biocidal properties of iodine, iodinated phenolic compounds prepared in an eco-efficient way by laccase catalysis could be useful as antifungal agents. Therefore, we investigated whether the products of laccase-iodide biotransformation inhibit the growth of basidiomycete and ascomycete fungi. In agreement with previous studies [27], [35] we found that the lignin-related non-iodinated educts vanillin and to a lesser extent also ethyl vanillin impaired the growth of basidiomycete fungi in a concentration-dependent manner (Table 3). The toxicity of phenolic compounds is thought to predominantly rely on their interaction with cell membranes, which in turn is related to their lipophilicity [36]. The microbial cell membrane acts as a selective permeability barrier between the cytoplasm and the cell's external environment which can be damaged by phenols, leading to the release of intracellular constituents [37]. Once released to the cytoplasm, phenols may also cause coagulation of soluble cytoplasmic constituents, leading to cell death or inhibition of cell growth [38]. Iodination of either vanillin or ethyl vanillin significantly increased the antifungal activity of these compounds (Table 3). This could both be due to a further increased lipophilicity and to the enzymatic and/or abiotic release of biocidal free iodine from the iodo-compounds (in the sense of a iodophor, [1]). The growth inhibitory effect of iodovanillin and iodo-ethyl vanillin was strongest for the white rot fungus *Trametes versicolor.* This observation correlated with a visible brownish color on tests plates containing the iodinated compounds, presumably due to enzymatic iodine release. *T. versicolor* is known to constitutively secrete oxidative enzymes [13], [39], [40]. This was also the case in our study, as strong peroxidase and laccase activity was detected on all malt agar plates inoculated with this species (Figure 5). At least for laccase we could show that the enzyme is in fact capable of catalyzing the backward reaction *in vitro*, i.e., releasing iodine from iodovanillin. In contrast to *T. versicolor*, no brownish color and neither laccase nor peroxidase activities were detected when the wood degrading basidiomycete *O. placenta* (syn. *Postia placenta*) was grown on malt agar test plates. This is in agreement with the lower sensitivity of this species and the other brown rot fungi tested towards iodovanillin and iodo-ethyl vanillin (Table 3). The observed growth impairment of brown rot fungi by iodovanillin and iodo-ethyl vanillin could be either due to general, membrane-associated toxic effects or to the release of low amounts of iodine from iodo-phenols by abiotic decay or intracellular enzymes. It has to be mentioned however, that when growing on wood, laccase and other oxidative enzymes are secreted by *O. placenta* and other brown rot fungi [41], [42]. This might explain why laccase-iodide treatment of spruce wood also was highly effective in preventing wood decay by these species and not only by *T. versicolor* [9]. Enzymatically produced reactive oxygen species (ROS) are thought to play an essential role in the initial attack of wood, both by brown and white rot fungi [43]. It is likely that ROS produced in the initial phase of fungal wood colonization attack iodo-phenols and release biocidal iodine in laccase-iodide treated wood.

The ascomycete species *A. pullulans* was not affected by vanillin and iodovanillin added to malt agar test plates in concentrations of up to 4 mM (Table 3). Possibly, this species does not produce any oxidative enzymes capable of releasing iodine from iodovanillin on nutrient-rich malt agar plates. Interestingly, laccase-iodide treatment protected wood also against colonization by *A. pullulans*, even after rigorous washing of the surface before inoculation [9]. A possible explanation for this effect is either the induction of the expression of secreted oxidative (e.g. cellulose-degrading, H_2_O_2_-releasing) enzymes on the exclusively polymeric growth substrate wood or the non-covalent binding of biocidal iodine species to the wood surface in the form of lignin-charge-transfer complexes or as cellulose-bound triiodide [44], [45].

The results obtained for *T. versicolor* indicate that phenolic compounds with covalently bound iodine can be considered enzyme-responsive, antifungal molecules. Enzyme-responsive materials (ERMs) are designed for undergoing specific, functional changes upon interactions with enzymes and receive increasing interest especially in the biomedical field [46]. Examples for antimicrobial ERMs are polyhexanide-filled hyaluronic acid nanocapsules which specifically respond to hyaluronidases secreted by pathogenic bacteria [47] or fusidic acid-loaded PLGA ultrafine fibers which show an accelerated release of the antibiotic when heavily colonized by *Staphylococcus aureus* [48]. We suggest that iodinated phenolic compounds and polymers (e.g. iodinated lignin) function as antifungal ERMs.

The observed *in vitro* iodination of phenolic compounds by laccase catalysis in the presence of iodide salts leaves the possibility that such reactions also occur in natural environments. Halogenated compounds are widespread in nature, although they comprise mostly chlorinated and brominated molecules [49], [50]. In marine environments, also organo-iodine compounds are found, e.g., the plakohypaphorines produced by Carribean sponges [51]. Laccases naturally present in soil were shown to mediate the sorption of iodide, presumably by iodination of soil organic matter, e.g. of polyphenolic humic acids [10]. An iodide-oxidizing multicopper oxidase was purified from a bacterium living in iodide-rich natural gas brine water [6], theoretically this enzyme could be involved in the iodination of phenolic compounds. The fact that laccase-like multicopper oxidases are found in numerous organisms of all domains of life, including marine bacteria [52], [53] also speaks in favor of naturally occurring iodination reactions mediated by such enzymes.

## Supporting Information

Figure S1 **HLPC-MS analysis of chemically synthesized 5-iodovanillin.** Upper panel: HPLC-UV chromatogram, lower panel: mass spectrum (negative mode). The compound was dissolved as obtained from the supplier in ethyl acetate to a final concentration of 1 mM.
(TIF)
