# Supplementary figures and images for: Laccase Catalyzed Synthesis of Iodinated Phenolic Compounds with Antifungal Activity
Source: PLoS One. 2014 Mar 3;9(3):e89924. doi: 10.1371/journal.pone.0089924 (PMC3940670; doi:10.1371/journal.pone.0089924)

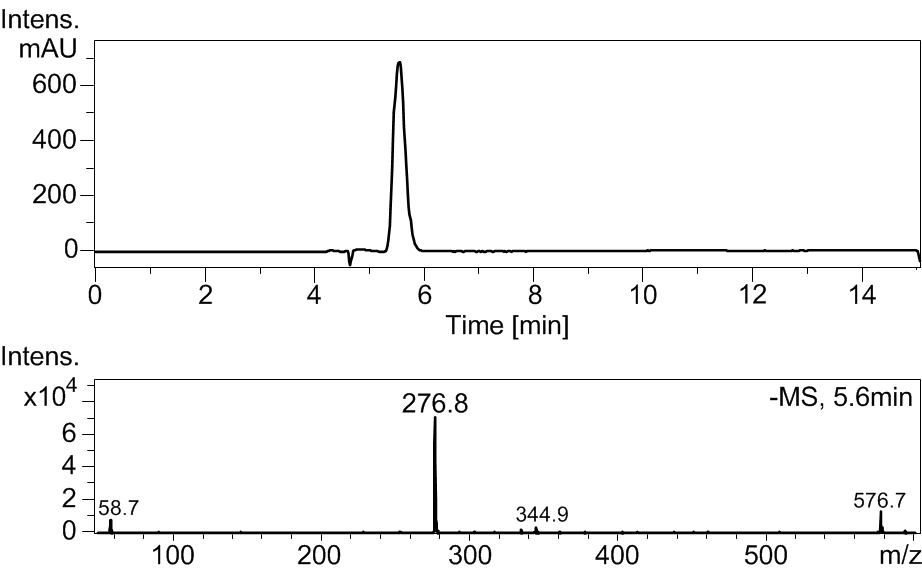

Supplement: Figure S1 — HLPC-MS analysis of chemically synthesized 5-iodovanillin. Upper panel: HPLC-UV chromatogram, lower panel: mass spectrum (negative mode). The compound was dissolved as obtained from the supplier in ethyl acetate to a final concentration of 1 mM. (TIF) [file pone.0089924.s001.tif]
